# Quantitative Ultrasound Changes in Echotexture and Functional Parameters after a Multicomponent Training Program in Pre-Frailty Individuals: A Pilot Randomized Clinical Trial

**DOI:** 10.3390/healthcare9101279

**Published:** 2021-09-28

**Authors:** Sergio López-López, Helios Pareja-Galeano, Jaime Almazán-Polo, Charles Cotteret, Patricia Téllez-González, César Calvo-Lobo, Luis Perea-Unceta, Carlos Romero-Morales

**Affiliations:** 1 Faculty of Sport Sciences, Universidad Europea de Madrid, 28770 Madrid, Spain; sergio.l.lopez@gmail.com (S.L.-L.); jaime.almazan@universidadeuropea.es (J.A.-P.); charles.cotteret@universidadeuropea.es (C.C.); ptous12@hotmail.com (P.T.-G.); carlos.romero@universidadeuropea.es (C.R.-M.); 2 Department of Sport Sciences, Universidad Autónoma de Madrid, 28049 Madrid, Spain; 3 Faculty of Nursing, Physiotherapy and Podiatry, Universidad Complutense de Madrid, 28040 Madrid, Spain; cescalvo@ucm.es; 4 Albertia Care Center, 28232 Madrid, Spain; lperea@albertia.es

**Keywords:** aging, frailty, image analysis, exercise, multicomponent intervention, ultrasound imaging

## Abstract

*Objective:* Currently, ultrasound imaging (USI) is considered a feasible tool in the evaluation of structural and textural muscle differences due to aging. The main aim of this study was to evaluate sonographic changes in muscular structure and function after a 12-week multicomponent training program in pre-frailty individuals. *Design:* A prospective, randomized, clinical trial was carried out. *Participants:* Thirty-two pre-frailty subjects were recruited and randomly divided into a multicomponent training program group (*n* = 16; Multicomponent group) and a conventional care group (*n* = 14; Control group) with a 12-week follow up. *Main outcome measures:* Rectus femoris thickness, cross-sectional area (CSA), echointensity, echovariation and vastus lateralis pennation angle tests were carried out to assess the structure and echotexture, and the force–velocity (F-V) profile for muscle power and muscle strength was employed to assess the functional parameters. *Results:* Statistically significant differences (*p* < 0.05) were shown for the left rectus femoris echointensity and in the functional parameter of muscle power after a 12-week program for the multicomponent training group compared to the conventional care group. *Conclusions:* Pre-frailty elderly subjects showed a decrease in rectus femoris echointensity (RF-EI) and an increase in the functional parameter of muscle power after a 12-week multicomponent training program compared to the control group.

## 1. Introduction

Geriatricians define frailty as a biological syndrome characterized by a decrease in biological reserve and resistance to stressors, resulting from alterations of several physiological systems [1]. Furthermore, frailty has been considered a highly prevalent condition in older adults and also related to an increase in the risk of falls, institutionalization, disability and mortality [2]. García García et al. [3] stated that pre-frailty affects up to 50% of the elderly population over 65 years old. In addition, Theou et al. [4] reported that more than 85% of pre-frailty elderlies need assistance at home or in care facilities, which decreases their functionality and quality of life. Physical deterioration (i.e., fatigue, decrease in the gait speed and grip strength), weight loss and reduced physical activity define the diagnoses of frailty [2]. In addition, one of the major pathophysiological factors associated with frailty is the loss of muscle mass induced by biological aging, such as sarcopenia [5].

The current literature suggests that older adults who practiced physical activity (i.e., strength training programs, walking), maintained healthy lifestyles and kept in touch with family and friends had a higher probability of living independently and an increased quality of life [5]. Several authors focused on the benefits of physical exercise for improving functional and cognitive capacities in order to delay the onset of frailty. For example, Yamada et al. reported significant improvements in frailty scores in individuals who participated in a multicomponent training program with respect to a control group after 1 year (frailty checklist: Exercise group, from 7.41 to 7.11; control group, from 7.34 to 8.02) [6,7]. Therefore, exercise programs, such as balance training, coordination training, endurance training, power training and multicomponent exercises, presented benefits in frail elderly populations.

Multicomponent exercise programs are composed of a range of physical activities based on strength, balance and flexibility interventions [5]. In addition, Bryne et al. [8] reported that interventions that comprise maximal intended movement velocity, easy training methods with low-loading exercises and low-frequency training could have benefits for the maintenance of functional performance in frailty populations. Cadore et al. [9] showed the effectiveness of a 12-week multicomponent exercise program on the functional condition and physical performance of elderly people. Regarding the short exercise programs, Losa-Reyna et al. [1] compared a 12-week supervised program of muscle power and walking based on high-intensity interval training (HIIT) with conventional care treatment, reporting an improvement in function and frailty in pre-frail individuals.

The use of ultrasound imaging (USI) to assess musculoskeletal and connective tissues has exponentially increased in recent years [10]. Furthermore, evaluations of the muscular cross-sectional area (CSA), thickness, pennation angle of the muscle fibers and fascial connective tissues were employed to complement physical therapy examination [11]. The use of USI to evaluate the lower limb muscle features has been employed in several populations, for example, Lin et al. [12] showed that the vastus medialis obliquus (VMO) morphology was related to the patellar alignment based on the VMO muscle fibers’ insertion in the patella. In addition, significant differences have been shown in the VMO architecture between individuals with and without patellofemoral pain syndrome by USI [13]. Regarding the vastus lateralis (VL), Ticicnesi et al. [14] presented a standardized protocol to assess the VL muscle size and architecture. In addition, several authors reported good reliability for USI muscle parameters. In this line, Raj et al. [15] showed good reliability for the use of USI to evaluate the VL in older adults. Seymour et al. [16] examined the rectus femoris (RF) muscle, reporting that ultrasound approaches were good radiation-free alternatives to quantify the CSA in patients with chronic obstructive pulmonary disease, due to the relationship between muscle loss and mortality. Furthermore, Berger et al. [17] noted that USI of the RF was a reliable and accurate method to evaluate muscular features in older people. Therefore, the most common quantitative features evaluated by USI are thickness, CSA and fascicle pennation angle. In this line, recent research has been focused on pixel gray-scale analysis to obtain a better understanding of the muscle architecture and connective tissue, which could be beneficial to assessing individuals with musculoskeletal disorders. Thus, several studies have previously reported muscle quality assessed through the quantification of echointensity (EI) with gray-scale analysis of the region of interest (ROI) [18,19]. In addition, EI changes have been employed to detect muscle variations in individuals with pathology, such as amyotrophic lateral sclerosis, with this being considered a potential prognostic biomarker of degenerative disease progression [20,21]. Nevertheless, a high inter-individual variability for EI limits the ability of this procedure as a diagnostic method or a reliable biomarker. In order to establish a disease biomarker not influenced by external factors, Rios et al. [22] employed echovariation (EV) to determine plantar fasciitis, describing this method as a reliable and easy-to-perform procedure. EV is a first-order statistical parameter used to describe the dispersion of the gray range from an average value, which offers information about tissue homogeneity [23,24]. In the interpretation of EI, the majority of research has employed a scale between 0 (black) and 256 (white) [25]. EV interpretation was intimately related to the formulae [EV = (SD/EI) × 100], thus bigger scores might be related to heterogeneous muscle tissues and, therefore, a healthy context [26]. However, more research on tissue homogeneity is still needed.

All assessments for pre-frailty elderly individuals who carried out multicomponent exercise programs have been focused on functional and cognitive parameters. Nevertheless, further research about muscle architecture is still needed in order to evaluate the morphological features and effectiveness of physical therapy programs. Thus, the primary aim of the present study was to investigate the effects of a 12-week multicomponent training compared to a conventional care program on RF thickness and CSA, VL pennation angle and muscular EI and EV parameters in pre-frailty elderly subjects, determined by USI. The force–velocity (F-V) profile was measured to assess muscle power and muscle strength in order to check the functional parameters among groups as a secondary aim. We hypothesized that USI parameters, such as thickness and imaging echotexture, could be useful to determine the effects of a 12-week multicomponent training in pre-frailty adults.

## 2. Methods

### 2.1. Study Design

A prospective, randomized, clinical trial was carried out on pre-frail elderly individuals over a 12-week period between September and December 2018 in Residencia Valle de la Oliva (Majadahonda, Spain). This study also adhered to the Consolidated Standards of Reporting Trials (CONSORT) guidelines.

### 2.2. Ethical Considerations

The ethics committee of Hospital Universitario de la Princesa, Madrid, Spain, approved this study. The study was also registered in the database clinicaltrials.gov (NCT03986840). Prior to the intervention, all participants signed the informed consent form. In addition, the present study was carried out in accordance with the ethical standards of the Declaration of Helsinki for human experimentation [27].

### 2.3. Participants

**Figure 1:**
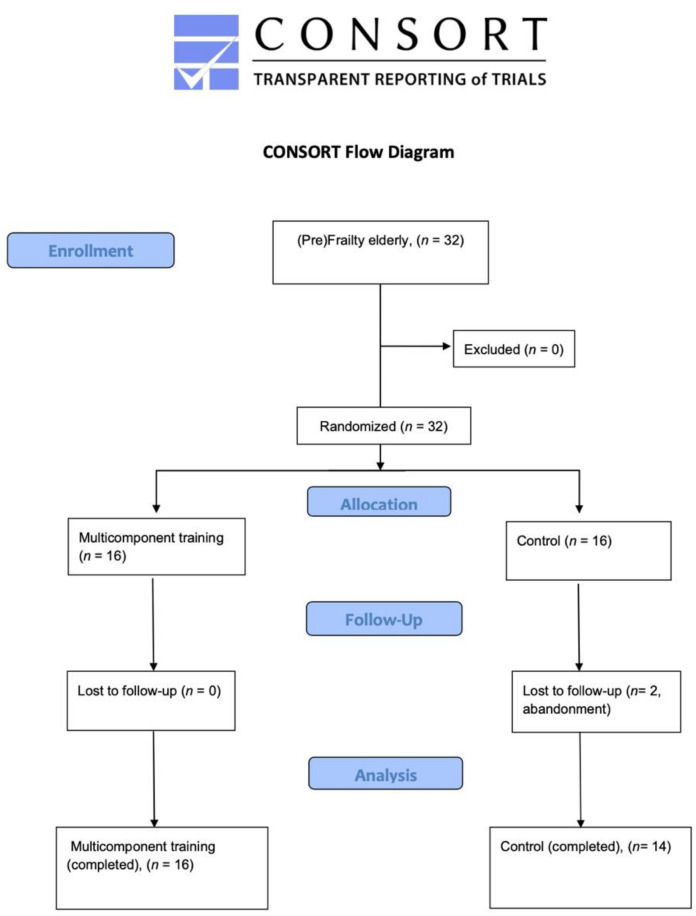
Flow chart diagram.

For this study, a sample of 32 participants was recruited in Residencia Valle de la Oliva (Majadahonda, Spain) and divided into a multicomponent training group (*n* = 16) and a control group (*n* = 14) (Figure 1). Participants’ inclusion criteria were subjects (males or females) considered as pre-frail [2], aged 75 years or more [1], subjects with a score between 2 and 10 points in the Short Physical Performance Battery (SPPB) [28] and subjects with the capability to walk either independently or assisted. Exclusion criteria were the absence of pre-frailty or frailty syndrome [5], a Barthel index for disability lower than 15 [1], cardiac arrest, cardiac failure, any unstable medical condition, any surgery in the 6 months preceding the beginning of the study and severe cognitive deterioration. 

### 2.4. Sample Size Calculation

G*Power software was employed to calculate the sample size with the main outcome measurement of Right RF-EI (mm) of a pilot study (*n* = 12) divided in 2 groups (mean ± SD): 6 subjects for the multicomponent training group (24.04 ± 5.18) and 6 individuals for the control group (19.32 ± 6.37). During the procedure for determining the sample size, an α error of 0.05, an effect size of 1.15 and a power of 0.80 with a 1-tailed hypothesis were employed. Finally, a total sample of 20 individuals was calculated. For the present study, we were able to recruit 32.

### 2.5. Randomization

Participants were randomized into a multicomponent training group and a control group using the free software randomized.org with a 1:1 ratio. Individuals were informed and reminded weekly not to discuss their randomization group with the USI evaluator. Nevertheless, the staff that carried out the physical intervention were aware of the participants’ assignment. 

### 2.6. Multicomponent Training Group

The 12-week multicomponent training program was carried out following Losa-Reyna et al.’s [1] guidelines, with a total of 36 training sessions distributed across 3 weekly sessions. Each session had a duration of 45 min with a minimum resting period of 48-h between sessions. At the start of the training session, all the individuals warmed up with 5-min of walking at the usual gait speed on a treadmill with 1% inclination (Treadmill TC-140, Domyos). Afterwards, participants carried out a leg press exercise (Bodytone Evolution Leg Press E59) and plantar flexion with a step platform following an interval-type cardiovascular exercise on a treadmill. For the leg press exercise, subjects performed 3–4 sets of 8–15 repetitions with 30–60% F_0_ (maximum muscle strength) with 1 to 3 min of recovery between sets. For the plantar flexion exercise, individuals carried out 3 sets of 4–12 repetitions at their bodyweight using 1 or 2 legs with 1 to 3 min of recovery between sets [1]. During the first two weeks, participants followed a conditioning period in order to integrate the training methodology (with special emphasis on exercise execution and development) with 3 sets of 12–15 repetitions at 30–40% F_0_ for the leg press and 6–8 repetitions for plantar flexion with their bodyweight with 1 to 3 min of recovery. Considering the interval-type cardiovascular exercise, the conditioning period consisted of 8 to 10 min of walking on a treadmill at 50–60% of their maximal gait speed. For muscle power performance, the subjects performed 4 sets of 8 repetitions at maximal intensity—due to the F-V profile—with the leg press, and for plantar flexion, 3 sets of 6–8 repetitions with 1 to 3 min for a recovery period.

### 2.7. Usual Care (Control) Group

Individuals assigned to the usual care group received normal outpatient care in a 45-min session based on joint mobility—cervical, upper and lower limb—with all the individuals seated in a chair. 

### 2.8. Outcome Measurements

Prior to developing the interventions, all USI assessments were carried out. In addition, on a separate day, the subjects were evaluated attending to their F-V profile for muscle power and muscle strength evaluation. At the end of the 12-week intervention period, all subjects were re-evaluated regarding USI and functional parameters. Thus, a total of 2 assessments were conducted across three separate days (day 1: USI evaluation; day 2: F-V and day 3: at 12-weeks follow up).

### 2.9. Ultrasound Imaging Evaluation

**Figure 2:**
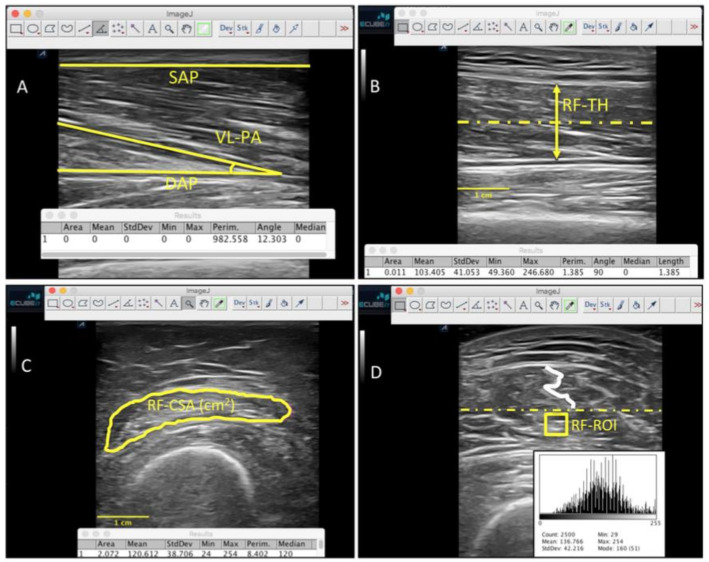
Ultrasound imaging measurements of the morphology and echotexture. (**A**), VL-PA at 65% of the distance between the greater trochanter and the intercondylar notch of the knee; (**B**), RF-TH at 50% of the distance between the anterior superior iliac spine and the superior edge of the patella; (**C**), RF-CSA at 65% of the distance between the anterior superior iliac spine and the superior edge of the patella; (**D**), RF-ROI at 50% of the distance between the anterior superior iliac spine and the superior edge of the patella for the extraction of echotextural variables (echointensity and echovariation). Abbreviations: DAP, deep aponeuroses; SAP, superficial aponeuroses; RF-CSA, cross sectional area of the rectus femoris at 65%; RF-TH, thickness of the rectus femoris at 50%; RF-ROI, range of interest of the rectus femoris; SAP, superficial aponeuroses.

All USI examination and imaging analyses were carried out by the same physiotherapist (J.A.P) with more than 5 years of experience and specialization, who was blinded for group assignment. A high-quality ultrasound system (Ecube i7; Alpinion Medical Systems, Seoul, Korea) with an 8 to 12 MHz linear transducer (Broadband Linear Array type L3_12T; 128 elements) was used to extract all ultrasound B-Mode images in both extremities. Firstly, patients were placed in the supine position at rest with the lower limb in full extension to determine the VL pennation angle (VL-PA) (pre-fixed preset using 5 cm of depth, 10 MHz frequency, 60-point gain, 60-point dynamic range and 1 focus located at 2.5 cm depth), following the protocol described by Ticinesi et al. [14] The probe was placed at 65% of the distance between the greater trochanter and the intercondylar notch of the knee. Then, the lateral and medial edges of the muscle were identified to establish 50% of this distance as the location point for image extraction. Secondly, bilateral images were obtained to assess the cross-sectional area of the rectus femoris (RF-CSA), the thickness of the rectus femoris (RF-TH) and the range of interest of echointensity of the rectus femoris (RF-EI) and echovariance (RF-EV) at 50% of the distance described above (pre-fixed preset using 4.5 cm depth, 10 MHz frequency, 60-point gain, 60-point dynamic range and 1 focus located at 2.5 cm depth). Thereby, for this exploration, participants stayed in a supine position at rest with a crib under the knee to maintain a position of 30° of flexion. Likewise, the probe was placed transversally at 50% (RF-EI and RF-EV) and 65% (RF-CSA) of the distance between the anterior superior iliac spine and the superior edge of the patella and vertically at 50% using the midpoint of the RF as a reference to determine the rectus femoris thickness (RF-TH) [20] (Figure 2).

A total of three images were captured at each location point and all measurements were developed limiting the pressure on the skin to decrease the echogenic artifacts produced by tissue compression [29]. After imaging extraction, gray-scale images of USI were saved in Digital Imaging and Communications in Medicine (DICOM) format and were analyzed on an offline computer using version 2.0 of ImageJ software (U.S.—National Institutes of Health; Betheseda, Maryland, USA) by the same researcher blinded to the allocation group (J.A.P.) and were calibrated from pixels to cm for USI measurements. Firstly, VL-PA was calculated drawing a projection line of the superficial and deep aponeuroses and using the insertion of the furthest right fiber of the distal aponeuroses as a reference for the angle assessment (VL-PA; Figure 2A). The mean of 3 repeated measurements of each image was used in order to increase the reliability [30]. Secondly, RF-CSA at 65% of the distance between the anterior superior iliac spine and the superior edge of the patella was measured using the inner edge of the connective tissue of the muscle (RF-CSA; Figure 2B). Thirdly, RF-TH at 50% was calculated at the midpoint between the anterior iliac spine and the superior aspect of the patella (RF-TH; Figure 2C) [31]. Finally, the echotexture of the tissue was evaluated by quantitative analysis of pixels on the RF at 50% displaying a histogram of the pixels’ distribution. Afterwards, EI and EV of the tissue were calculated counting 2500 pixels (50 × 50) in a rectangular range of interest (RF-ROI; Figure 2D) placed in the center of the RF, below its central aponeuroses, in order to reduce the increase in reflection from hiperechogenic connective tissue, at 1 cm deep using the ultrasound devices’ scale bar from the lateral side of the extracted images as a reference [20,23,32,33,34]. Echointensity was registered as the mean value of the ROI histogram while EV was calculated using the previous method described by Ríos et al. that uses the standard deviation (SD) and mean value of intensity (EI) in the ROI (EV = SD/EI × 100) [23]. In addition, the mean of three repeated values for each ROI was also employed to calculate EV and EI [23,33].

### 2.10. Functional Parameters Assessment

Before functional testing, all individuals attended 2 familiarization sessions in order to ease the proper execution of the exercise. According to Alcázar et al.’s [35] procedure, for the F-V and muscle power evaluation, individuals performed a 5 min cycling (Cicloergometer Domyos E Seat) warm up with a light intensity (20–40 W). In addition, the participants performed 3 sets of 10 repetitions on the leg press with an intensity corresponding to 40% of the individual’s body mass with 1-min recovery periods [35]. The participants were also instructed to perform the leg press with a range of motion (ROM) of 90–100° with the hip and knee joints, to full extension. During the concentric phase, F-V was assessed with a linear transducer device (T-Force, Ergotech, Murcia, Spain) [1]. All the subjects were instructed to perform each repetition as fast and strong as possible. Data recorded were muscle force and the mean of the highest velocity, discarding those repetitions that were not performed at the maximal speed [1]. Muscle strength and muscle power were extracted from the recorded data following the F-V regression formula reported by Alcázar et al. [35].

### 2.11. Statistical Analysis

SPSS 23.0 software (IBM SPSS Statistics, Armonk-NY; IBM-Corp) was employed for the statistical analysis. First, the Shapiro–Wilks test was used to assess the normality of the data distribution. Second, the Student t-test for independent samples was used considering the homogeneity of variance using Levene’s test for baseline comparisons. Third, to assess the effects of intra-subject (pre and post) and inter-subject (treatment groups) values on the dependent variables, a two-way analysis of variance (ANOVA) for repeated measures was performed. The level of significance was set at *p* < 0.05 with an α error of 0.05 (95% confidence interval) and the desired power of 80% (β error of 0.2).

## 3. Results

Sociodemographic data parameters did not show significant differences between the multicomponent training and control groups (Table 1). Left RF-EI showed a significant decrease (*p* < 0.05) at 12 weeks in both groups and also reported significant differences (*p* < 0.05) in the multicomponent group after the intervention compared to controls. Right RF-EI, Right RF-EV, Left RF-EV and Left RF-EI showed significant differences (*p* < 0.05) in both groups at 12 weeks. However, no significant differences between groups were found (Table 2). The rest of the USI variables did not show significant differences (*p* > 0.5). Regarding the functional parameters, muscle power showed a significant increase (*p* < 0.05) at 12 weeks in both groups, and between groups in favor of the multicomponent training group. Nevertheless, the muscle strength parameter did not show significant differences. No deleterious effects have been reported as an effect of long-term treatment.

## 4. Discussion

The purpose of this study was to conclude if pre-frail individuals who participated in a 12-week multicomponent training showed differences in RF-TH, RF-CSA, RF-EI, RF-EV and VL-PA compared to participants who participated in a conventional care intervention (control group). According to the authors’ knowledge, this is the first experimental study that compares sonographic changes in pre-frail subjects among a multicomponent exercise program group and a control group. These variables have been considered of interest in the study of sonographic muscle architecture and echotexture, as well as the ability to predict the degree of alteration in several pathologies or degenerative conditions [16,17,18,19].

The main findings of this study, after a 12-week intervention with a multicomponent exercise program, reported benefits in a functional parameter, namely muscle power, and changes in RF-EI compared to the control group, whereas RF-EV showed significant differences in both groups over time. Nevertheless, structural USI measurements of RF-TH, RF-CSA and VL-PA did not show significant differences after the intervention. In this sense, although age-related muscle mass decline and structural modifications of muscle architecture are considered one of the main determinants in aging [36,37,38], there is still some controversy about the relationship between changes observed in some USI variables and functional parameters. According to this, Abe et al. [38] reported poor correlation between functional parameters (e.g., gait speed) and muscle thickness of their thigh diagnosed by ultrasound. Similar results have also been observed by Strasser et al. [37], who did not find a relationship between the muscle pennation angle and the maximum voluntary muscle strength in quadriceps femoris. These authors also reported low inter-intra operator reproducibility of pennation angle measurements, which is considered an important limitation in USI examination of the elderly population due to aging degenerative alterations. In contrast, Losa-Reina et al. [1] reported benefits in functional parameters with a 6-week multicomponent training in physical performance in elder patients. In addition, the muscle thickness anterior/posterior ratio, assessed by ultrasound imaging in the RF, has been proposed as an early biomarker of sarcopenia in older adults [39]. Likewise, prior research showed muscle thickness decreases in individuals with muscular atrophy (i.e., patients with amyotrophic lateral sclerosis) [20,40], and in a similar way, the decrease in rectus femoris cross-sectional area presented a strong association with the mortality index, length of stay and readmissions in patients with COPD [41]. Despite the diversity of results, ultrasound has been reported as a feasible tool to predict muscle strength changes that befall faster than muscle mass decline [42].

### 4.1. Quantitative Ultrasound Imaging for Tissue Echogenicity and Homogeneity

Regarding muscle echogenicity to evaluate tissue homogeneity related to metabolic derangements, inflammation, fibrosis or intramuscular fat infiltration, our results showed a decreased EI in both groups, which could be interpreted as a change in the intramuscular content [43,44]. Major differences were observed in the intervention group compared to the control, but improvements in both could be explained by the increase in training levels and maintenance of physical activity, respectively. Specifically, muscle echointensity has been proposed as a possible biomarker to predict frailty, detecting intramuscular adipose tissue infiltration or “myoesteatosis”, as well as identifying intramyocellular lipid droplet characteristics in degenerative conditions such as sarcopenia, neurodegenerative diseases or myopathies [19,23,33,45,46]. Similar findings were reported by previous authors who considered EI a useful parameter in assessing intramuscular content changes with aging; however, no previous studies have researched the differences between pre-frail groups after a multicomponent intervention [24,47,48]. Fukumoto et al. [47] found a negative correlation between muscle strength and RF-TH with RF-EI in healthy young people, without correlation with subcutaneous fat thickness, percentage of body fat or body mass index. In such a way, previous studies have found a negative correlation between RF-EI and muscle strength in elderlies [49], exhibiting better results in pre-frail elderlies when compared with different levels of frailty between similar ages [48].

Significant differences between groups were not observed in RF-EV; nevertheless, our results exhibited a light tendency of increased EV in all groups of study, slightly more marked in the multicomponent training group. EV quantifies the level of deviation of gray pixels from the mean, being proposed as a variable that evaluates tissue homogeneity [17,37]. Thus, EV was described as a possible biomarker related to muscle strength and disability in amyotrophic lateral sclerosis (ALS), where muscles affected by these conditions displayed higher values indicating greater heterogeneity [23]. Despite the trend of our results and their agreement with previous research, there is still a lack of interpretation of EV and the results could vary depending on the stage of the degenerative disease [24].

### 4.2. Future Studies and Clinical Implications

Our results suggest that the main differences have been observed in echogenicity (RF-EI) and muscle power force in pre-frailty participants after completing a multicomponent training program. Thus, differences in EI and muscle power between pre-frailty groups after a physical activity intervention might have clinical importance, placing EI as a possible useful biomarker in the evolutionary control of ultrasound changes in muscle content after physical exercise. Despite the fact that significant differences were not shown in architectural ultrasound variables, we believe that these results could be explained by the lower biological potential of the elderly population to express architectural muscle tissue changes due to aging effects. Therefore, this relationship between tissue echogenicity and muscle power force could be inversely similar to the hypothesis associated with early reduction of muscle strength before observing changes in muscle mass [23]. Hence, more studies are needed to better understand the role of EV and EI in aging, as well as its clinical application, offering information on tissue homogeneity echogenicity modifications through physical activity intervention. Future studies should consider the use of second-order texture analysis, such as the gray-level co-occurrence matrix (GLMC), that provide information about gray-level patterns investigating the relationship with neighboring pixel intensities [5].

## 5. Limitations

Some limitations must be considered regarding this study. Firstly, ultrasound measurement of CSA at 65% of the anterior thigh distance described in the method reflected different patterns between subjects due to the variability observed in the height at which the rectus femoris was found. CSA at 50% of the distance was not registered due to the limitation of the probe size. Secondly, the intrinsic limitations associated with the study population showed difficulties during the displacement of the study subjects that could affect the reliability of ultrasound measurements, especially during the evaluation of VL-PA due to moderate cognitive impairments of some participants. In addition, the development of all the exercises sitting on a chair for the control group was also considered a limitation. Nevertheless, all measurements were collected in the same supine position to reduce displacement of the participants. Thirdly, an intra-class reliability assessment was not performed. Moreover, the extended field image mode has not been employed. Finally, despite its promising role as a biomarker, the parameters of echotexture evaluation are troublesome measurements that should be interpreted with caution and must be carried out by an expert in ultrasonography with specific training using a closed-design protocol to improve reproducibility [48]. Moreover, the findings of the present study should be interpreted considering the limited sample size.

## 6. Conclusions

Pre-frailty elderly subjects who carried out a 12-week multicomponent training program exhibited a decrease in RF-EI and an increase in the functional parameter of muscle power compared to those who received conventional care. No differences were found in USI measurements evaluating the muscular structural component (RF-TH, RF-CSA, VL-PA), which could indicate a greater interest in the evaluation of echotextural changes in this population subgroup as a control parameter of treatment. Nevertheless, these findings should be interpreted with caution due to the variability of the measurement procedures during USI evaluation as a result of the lack of more standardized protocols.

## Figures and Tables

**Table 1:** Sociodemographic data of the sample.

Data	Multicomponent (*n* = 16)	Control (*n*= 14)	*p*-Value Cases vs. Controls
Age, y	85.87 ± 7.18	86.57 ± 5.82	0.775
Mass, kg	64.70 ± 8.16	62.60 ± 13.16	0.599
Height, m	1.53 ± 0.12	1.53 ± 0.12	0.756
BMI, kg/m^2^	27.69 ± 3.62	26.14 ± 4.74	0.321

Abbreviations: BMI, body mass index.

**Table 2:** Vastus lateralis pennation angle, rectus femoris thickness, cross-sectional area, echointensity, echovariance, muscle power and muscle strength intrasubject effects.

			Intrasubject Effects	
Measure	Control *n* = 13	Multicomponent *n* = 16	Time Value F (Df); P (Eta^2^)	Treatment X Time F (Df); P (Eta^2^)
Right VL-PA				
Baseline	12.0 ±3.7	12.3 ± 3.8	F (1, 27) = 0.882; P = 0.356 (0.032)	F (1, 27) = 1.672; P = 0.207 (0.058)
12-weeks	10.8 ± 3.3	12.5 ± 2.1		
Left VL-PA				
Baseline	11.4 ± 2.1	14.1 ± 5.7	F (1, 27) = 2.136; P = 0.155 (0.073)	F (1, 27) = 0.277; P = 0.603 (0.010)
12-weeks	10.7 ± 4.0	12.7 ± 3.0		
Right RF-CSA				
Baseline	2.5 ± 1.1	2.8 ± 1.2	F (1, 27) = 0.020; P = 0.888 (0.001)	F (1, 27) = 0.475; P = 0.497 (0.017)
12-weeks	2.4 ± 0.7	3.0 ± 0.8		
Left RF-CSA				
Baseline	2.5 ± 0.8	2.9 ± 1.3	F (1, 27) = 0.006; P = 0.938 (0.001)	F (1, 27) = 1.029; P = 0.319 (0.037)
12-weeks	2.6 ± 0.7	2.8 ± 0.9		
Right RF-TH				
Baseline	1.0 ± 0.2	1.2 ± 0.2	F (1, 27) = 0.417; P = 0.524 (0.015)	F (1, 27) = 0.978; P = 0.331 (0.035)
12-weeks	1.0 ± 0.2	1.2 ± 0.2		
Left RF-TH				
Baseline	1.1 ± 0.4	1.2 ± 0.3	F (1, 27) = 0.763; P = 0.390 (0.027)	F (1, 27) = 0.386; P = 0.539 (0.014)
12-weeks	1.0 ± 0.3	1.2 ± 0.2		
Right RF-EI				
Baseline	121.5 ± 26.4	120.8 ± 16.9	F (1, 27) = 4.720; P = 0.039 (0.154)	F (1, 27) = 1.656; P = 0.209 (0.060)
12-weeks	118.5 ± 19.8	108.8 ± 17.3		
Left RF-EI				
Baseline	123.6 ± 24.8	130.4 ± 11.4	F (1, 27) = 21.856; P = 0.001 (0.457)	F (1, 27) = 6.862; P = 0.014 (0.035)
12-weeks	118.0 ± 22.7	110.3 ± 15.8		
Right RF-EV				
Baseline	24.9 ± 5.3	20.0 ± 6.3	F (1, 27) = 5.230; P = 0.031 (0.167)	F (1, 27) = 1.174; P = 0.288 (0.043)
12-weeks	26.7 ± 6.9	24.8 ± 9.4		
Left RF-EV				
Baseline	24.6 ± 6.8	21.9 ± 4.7	F (1, 27) = 7.926; P = 0.009 (0.234)	F (1, 27) = 0.677; P = 0.418 (0.025)
12-weeks	28.2 ± 6.1	23.9 ± 6.6		
Muscle Power				
Baseline	168.27 ± 64.1	161.0 ± 62.3	F (1, 27) = 19.645; P = 0.005 (0.278)	F (1, 27) = 9.321; P = 0.005 (0.272)
12-weeks	168.5 ± 52.9	195.9 ± 48.0		
Muscle Strenght				
Baseline	534.5 ± 210.1	552.5 ± 145.2	F (1, 27) = 1.569; P = 0.222 (0.059)	F (1, 27) = 1.352; P = 0.256 (0.051)
12-weeks	592.7 ± 224.5	554.6 ± 166.4		

Values are mean ± SD unless otherwise indicated. Abbreviations: RF-CSA, cross sectional area of rectus femoris; RF-EI, range of interest echointensity of rectus femoris at 50%; RF-EV, range of interest echovariance of rectus femoris at 50%; RF-TH, thickness of the rectus femoris; VL-PA, vastus lateralis pennation angle.

## Data Availability

The data presented in this study are available on request from the corresponding author. The data are not publicly available due to ethical condition.
